# Effects of Adjunct Antifibrotic Treatment within a Regenerative Rehabilitation Paradigm for Volumetric Muscle Loss

**DOI:** 10.3390/ijms24043564

**Published:** 2023-02-10

**Authors:** Jessica M. Motherwell, Connor P. Dolan, Sergey S. Kanovka, Jorge B. Edwards, Sarah R. Franco, Naveena B. Janakiram, Michael S. Valerio, Stephen M. Goldman, Christopher L. Dearth

**Affiliations:** 1 DoD-VA Extremity Trauma and Amputation Center of Excellence, Montgomery, MD 20815, USA; 2 Department of Surgery, Walter Reed National Military Medical Center, Uniformed Services University of the Health Sciences, Montgomery, MD 20815, USA; 3 The Henry M. Jackson Foundation for the Advancement of Military Medicine, Inc., Montgomery, MD 20817, USA

**Keywords:** trauma, extremities, skeletal muscle, fibrosis, regenerative medicine

## Abstract

The use of a rehabilitation approach that promotes regeneration has the potential to improve the efficacy of pro-regenerative therapies and maximize functional outcomes in the treatment of volumetric muscle loss (VML). An adjunct antifibrotic treatment could further enhance functional gains by reducing fibrotic scarring. This study aimed to evaluate the potential synergistic effects of losartan, an antifibrotic pharmaceutical, paired with a voluntary wheel running rehabilitation strategy to enhance a minced muscle graft (MMG) pro-regenerative therapy in a rodent model of VML. The animals were randomly assigned into four groups: (1) antifibrotic with rehabilitation, (2) antifibrotic without rehabilitation, (3) vehicle treatment with rehabilitation, and (4) vehicle treatment without rehabilitation. At 56 days, the neuromuscular function was assessed, and muscles were collected for histological and molecular analysis. Surprisingly, we found that the losartan treatment decreased muscle function in MMG-treated VML injuries by 56 days, while the voluntary wheel running elicited no effect. Histologic and molecular analysis revealed that losartan treatment did not reduce fibrosis. These findings suggest that losartan treatment as an adjunct therapy to a regenerative rehabilitation strategy negatively impacts muscular function and fails to promote myogenesis following VML injury. There still remains a clinical need to develop a regenerative rehabilitation treatment strategy for traumatic skeletal muscle injuries. Future studies should consider optimizing the timing and duration of adjunct antifibrotic treatments to maximize functional outcomes in VML injuries.

## 1. Introduction

Volumetric muscle loss (VML) was operationally defined by US military surgeons to describe the frank loss of skeletal muscle tissue that leads to chronic functional deficits, and has since been found to be highly prevalent among combat-related extremity trauma [1,2]. Within the last decade, many discoveries have been made in regard to understanding the underlying pathobiology of VML injuries, including a robust fibrotic host response via the transforming growth factor-β (TGFβ) pathway, which is believed to hinder skeletal muscle regeneration [3,4]. This process leads to excessive collagen deposition and the accumulation of myofibroblasts in the injured area, which is aberrant to canonical skeletal muscle repair and regeneration processes.

Numerous attempts have been made to develop treatment strategies that aim to attenuate the intrinsic fibrotic response in VML, making the wound microenvironment more permissive to myogenesis. These studies range from pharmaceutical to cell-based treatments and have been studied in both small and large preclinical animal models [5,6,7]. One example in particular, the small molecule pharmaceutical losartan, is an FDA-approved angiotensin II receptor antagonist that acts to dampen the downstream effects of the TGFβ pathway, which is a key player in the fibrotic cascade [8]. Losartan was originally developed as an anti-hypertensive treatment, but has also been demonstrated to elicit antifibrotic effects in the heart [9,10], kidneys [11], liver [12,13], and skeletal muscle (including rodent models of traumatic muscle injuries) [6,8,14,15]. The administration of losartan throughout the subacute period (i.e., 14 days) of a VML injury has been demonstrated to reduce fibrosis in a preclinical rodent model by day 28 post-injury [6]; however, the potential benefit of its chronic delivery (≥56 days) has yet to be established. This gap in knowledge is particularly important since VML injuries may benefit from long-term antifibrotic treatment, as collagen deposition and fibrotic scar formation are known to continue to develop and mature due to the prolonged and heightened expression of TGFβ, resulting in increased muscle stiffness [16]. The use of losartan in VML injuries is attractive because it is an FDA-approved drug that has been on the market for almost 30 years, has been demonstrated to be safe for chronic daily use, and thus has the potential for rapid clinical translation [17,18,19].

The use of rehabilitation in the treatment of VML is based on the plasticity of the remaining and/or regenerating muscle to adapt to mechanical stimulation in order to restore end-organ function and ultimately recover from injury. Currently, there is no clinical standard of care for rehabilitation after VML, in part due to the complex and highly variable nature of these injuries [20]. Thus, there is a critical need to improve our scientific understanding of how to effectively integrate rehabilitation into the treatment of VML. Physical activity, such as exercise-based rehabilitation, following a VML injury is intended to improve the strength of the residual musculature, increase range of motion, and enhance limb utilization [21]. In preclinical research studies, the use of voluntary wheel running as an exercise-based rehabilitation therapy has been successfully utilized for decades in a variety of small animal models. In the context of VML, voluntary wheel running has demonstrated a reduction in fibrosis at the defect site when used in conjunction with an autologous muscle graft, mitigated force imbalance in synergist muscles, and enhanced innervation following the implantation of an engineered scaffold [22,23,24]. Additionally, voluntary wheel running has been demonstrated to increase the number of satellite cells without affecting the muscle fiber cross-sectional area (i.e., inducing hypertrophy) [25]. Therefore, exercise-based rehabilitation therapy may enhance the efficacy of pro-regenerative treatments by creating a more favorable microenvironment for the existing musculature to repair and regenerate.

There has been a recent push towards integrating the fields of regenerative medicine and rehabilitation, a concept known as regenerative rehabilitation, with the intent of maximizing treatment outcomes for debilitating injuries [26,27,28]. Many efforts have been made in regenerative medicine to develop treatments for VML that can repair histologically appropriate skeletal muscle and restoring end organ function. These pro-regenerative endeavors have included cellular and acellular scaffolds [29,30,31,32], engineered hydrogels [33,34,35], autologous muscle grafts [22,36,37], and numerous others. However, given the enormous challenge of regenerating healthy skeletal muscle following VML injury, most of these interventions have only been partially successful in restoring form and function [38,39]. Recovery from traumatic injuries such as VML requires chronic clinical care that continues well past the implantation of a regenerative therapeutic. Thus, the need to implement a more comprehensive treatment strategy that considers not only the regeneration of damaged tissue, but also the long-term recovery process involving rehabilitation, is vital. The conceptual benefit of a regenerative rehabilitation approach is the potential to enhance the effectiveness of a pro-regenerative treatment while maximizing muscle function [27]. Physical activity may help the regenerative therapeutic (e.g., minced muscle graft [MMG]) to integrate with the surrounding tissue and potentially strengthen neuronal synapses, which are critical for organ function [26]. However, the science that will elucidate the specific biologic underpinnings of regenerative rehabilitation-based approaches is still ongoing and comprehensive evaluation is needed to optimize functional outcomes for VML injuries.

The purpose of this study was to evaluate the ability of an adjunct antifibrotic and rehabilitative treatment to enhance the efficacy of a pro-regenerative therapeutic for restoring form and function following a VML injury. This was performed using a well-established rodent model of the VML injury, where all animals received an MMG pro-regenerative therapeutic, then were randomly divided into four experimental groups: (1) vehicle treatment without rehabilitation (VEH-SED), (2) losartan treatment without rehabilitation (LOS-SED), (3) vehicle treatment with rehabilitation (VEH-RUN), or (4) losartan treatment with rehabilitation (LOS-RUN). It was hypothesized that a synergistic effect between the antifibrotic intervention (losartan) and rehabilitation strategy (voluntary wheel running) would facilitate an optimal microenvironment for a pro-regenerative therapeutic (MMG) to improve muscle function by reducing fibrotic deposition and increasing muscle strength, compared to either the antifibrotic or rehabilitative treatment alone with a pro-regenerative therapy. The development of a clinically relevant regenerative rehabilitation treatment strategy has the potential to improve functional outcomes beyond those achieved with a regenerative therapeutic or rehabilitation alone, which could improve the quality of life for those affected by VML injuries.

## 2. Results

### 2.1. Subcutaneous Losartan Delivery Reduces Fibrosis

**Figure 1:**
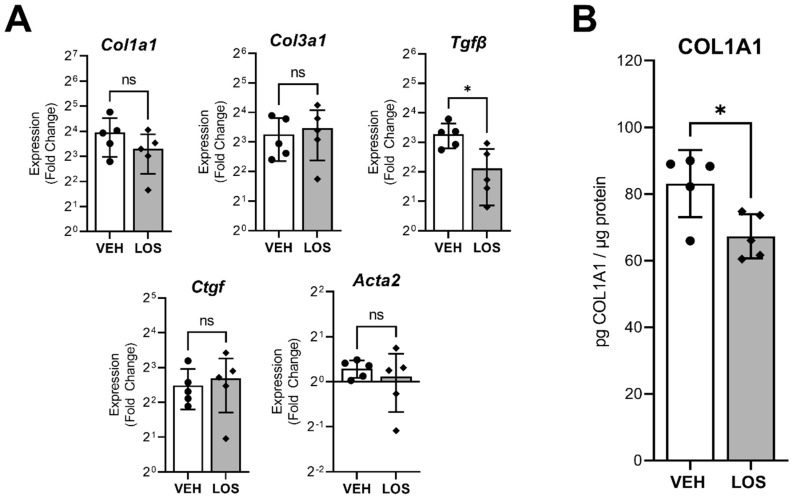
Evaluation of subcutaneous losartan delivery by gene expression and protein analysis at 28 days post-injury. (**A**) Transcriptional analysis of pro-fibrotic markers within a VML injury treated with either losartan (LOS) or vehicle (VEH). Bar graphs depict fold change in gene expression relative to vehicle uninjured limbs. (**B**) Quantification of COL1A1 protein levels produced in VML injured limbs by 28 days post-injury in losartan-treated (LOS) or vehicle-treated (VEH) groups. Data are presented as mean ± SD; n = 5 per group. The * indicates a significant difference of *p* < 0.01 by two-tailed Student’s *t*-test. “ns” indicates no significant difference (*p* > 0.05).

Losartan was subcutaneously delivered in VML-injured animals (without rehabilitation or MMG treatment) up to 28 days post-injury (DPI). The transcriptional analysis of pro-fibrotic markers showed a decrease in *TGFβ* (*p* = 0.013) when comparing the losartan treatment to vehicle controls (Figure 1A). However, no differences were observed for *Col1a1*, *Col3a1*, *Ctgf*, or *Acta2*. The quantification of COL1A1 protein levels showed a decrease (*p* = 0.019) between losartan and vehicle-treated limbs (Figure 1B), thus suggesting that by 28 DPI, the subcutaneous losartan administration is damping the collagen deposition following VML.

### 2.2. Assessment of Body and Muscle Weights

The impact of the regenerative rehabilitation treatment on the body weight of VML-injured animals and hindlimb muscle wet weights were investigated (Table 1). No differences were found between groups for the VML defect wet weights. Endpoint body weights were different as a function of both the rehabilitation and antifibrotic treatment, with no observed interaction between these variables. Specifically, animals assigned to voluntary wheel running gained less weight than their sedentary counterparts (Main Effect; *p* < 0.001), and interestingly, losartan-treated animals gained the least amount during the study (Main Effect; *p* < 0.001), where the VEH-RUN group on average gained 53% more weight than the LOS-RUN group. For the VML defect and injured tibialis anterior (TA) and extensor digitorum longus (EDL) wet weights, no differences were observed between groups. Contralateral TA and EDL wet weights were different as a function of rehabilitation (Main Effect; *p* = 0.007 and *p* = 0.006, respectively), where uninjured muscles within the voluntary wheel running groups were overall heavier compared to sedentary groups.

### 2.3. Evaluation of Voluntary Wheel Running

**Figure 2:**
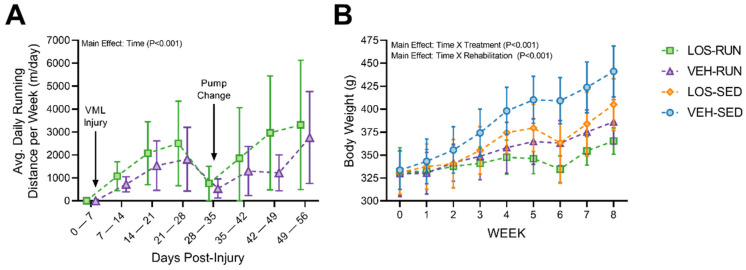
Running wheel and body weight evaluation over the 56 DPI time course of the study. (**A**) Daily running distances for each animal were averaged per week for LOS-RUN and VEH-RUN experimental groups. A gradual increase in daily running was observed; with the exception of the week injury was induced and osmotic pumps were explanted. Data are presented as mean ± SD; n = 7 rats per group. Differences were considered significant when *p* < 0.05 by fitting a Mixed-Effects model and the Holm-Sidak post hoc method. (**B**) Body weights were measured each week for all animals in the sedentary and running experimental groups. Animals gained weight throughout the duration of the study, as expected, with the running rehabilitation groups having the least increase. Data are presented as mean ± SD; n = 7–8 rats per group. Differences were considered significant when *p* < 0.05 by a three-way ANOVA followed by the Holm–Sidak post hoc test.

Each animal’s running distance (Figure 2A) and body weight (Figure 2B) were measured weekly throughout the study to assess any effects of voluntary wheel running. No differences were found between losartan and vehicle treatment groups for the average daily running distance; however, an expected increase in distance was observed as a function of time (Main Effect; *p* < 0.001) up to the 56 DPI study endpoint. Notably, we observed a decline in the running wheel activity at the 35 DPI time point (Figure 2A) following the explant and re-implantation of new osmotic pumps. Running wheel activity steadily increased and exceeded the prior average daily running distance over the subsequent following weeks. As expected, animal body weight increased over the duration of the study for all groups (Figure 2B). We also found a significant interaction between the timepoint and antifibrotic treatment (Interaction; *p* < 0.001) as well as an interaction between timepoint and rehabilitation (Interaction; *p* < 0.001). Overall, animals gained weight in all four treatment groups, with the VEH-SED treatment maintaining the highest weekly weight gain up to the 56 DPI endpoint.

### 2.4. In Vivo Neuromuscular Function

**Figure 3:**
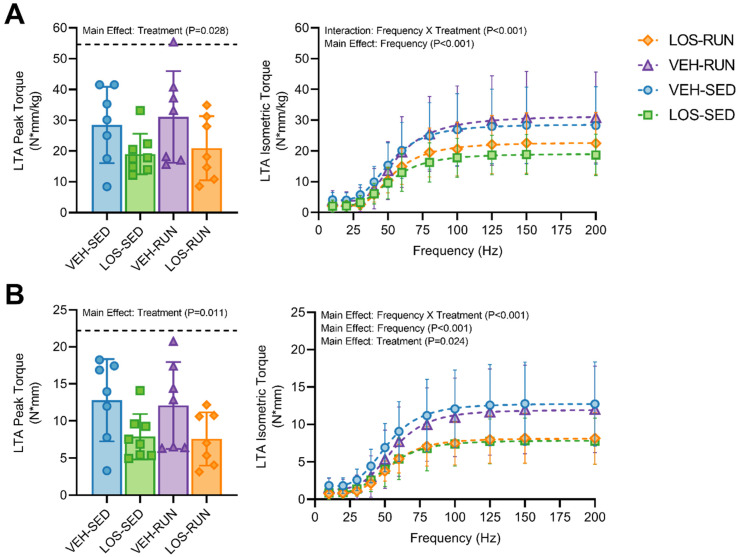
In vivo neuromuscular functional assessment. Evaluation of neuromuscular functional capacity in VML injured limbs was performed at 56 DPI. Peak torque and isometric torque as a function of frequency was evaluated and either normalized to body weight (**A**) or presented non-normalized (**B**). The black-dashed line denotes the average peak torque of the pooled contralateral uninjured limbs. Data are presented as mean ± SD; n = 7–8 rats per group. For LTA peak torque, differences were considered significant when *p* < 0.05 by a two-way ANOVA followed by the Holm–Sidak post hoc test. For the isometric torque as a function of frequency, differences were considered significant when *p* < 0.05 by a three-way ANOVA followed by the Holm–Sidak post hoc test.

Neuromuscular functional capacity was evaluated in VML-injured limbs at 56 DPI (Figure 3). An analysis of the maximal isometric torque normalized to endpoint body weights (Figure 3A) revealed a decrease by treatment (Main Effect; *p* = 0.028), but not by rehabilitation (Main Effect; *p* = 0.599), suggesting that the chronic losartan treatment has a negative effect on the neuromuscular functional output at 56 DPI. The peak isometric torque for uninjured contralateral limbs (Figure 3A, dashed line) was evaluated to elucidate the systemic effects of treatment and rehabilitation. As expected, increased levels of muscle function were observed in the uninjured limbs (Main Effect; *p* < 0.001) relative to the injured, with no differences observed between groups in the post hoc analysis. The additional evaluation of the isometric torque at increasing stimulation frequencies (force-frequency) was performed on VML-injured limbs (Figure 3A). The data revealed an interaction between the frequency and treatment variables (Main Effect; *p* < 0.001), where the losartan-treated groups’ isometric torque output consistently remained lower than the vehicle counterparts, regardless of rehabilitation.

### 2.5. Assessment of Fibrotic and Myogenic Proteins

**Figure 4:**
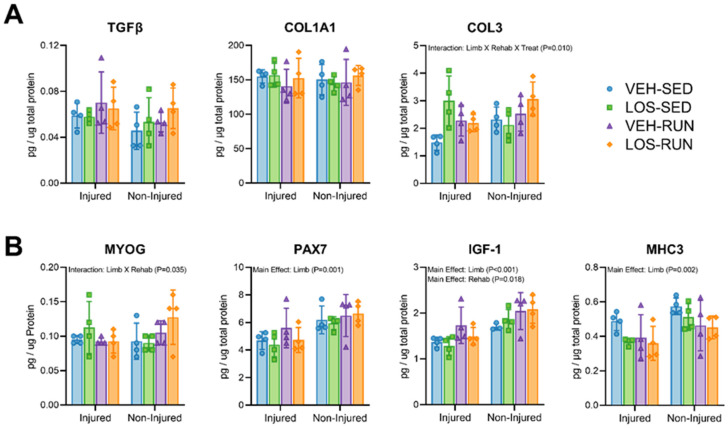
Pro-myogenic and pro-fibrotic protein quantification from injured and uninjured contralateral control limbs. Evaluation of pro-fibrotic (**A**) and pro-myogenic (**B**) protein presence in VML-injured and non-injured limbs was analyzed at the 56 DPI endpoint. No significant differences were observed for pro-fibrotic proteins, with the exception of an interaction for COL3. Pro-myogenic proteins displayed an overall increased trend in the non-injured limbs, and rehabilitation had a significant positive effect on IGF-1. Data are presented as mean ± SD; n = 3–4 rats per group. Differences were considered significant when *p* < 0.05 by fitting a Mixed-Effects model and the Holm–Sidak post hoc method.

The presence of proteins associated with fibrotic (Figure 4A) and myogenic (Figure 4B) processes in VML-injured and uninjured contralateral limbs was measured by ELISA assays. No differences in TGFβ or COL1A1 proteins were observed in the injured and uninjured limbs at the 56 DPI timepoint. For COL3 protein, there was an interaction between treatment, rehabilitation, and limb (Interaction; *p* = 0.010), where the uninjured limb protein concentration was increased compared to the injured, with the exception of the LOS-SED group.

With regard to proteins associated with myogenic processes, PAX7 (Main Effect; *p* = 0.001), IGF-1 (Main Effect; *p* < 0.001), and MHC3 (Main Effect; *p* = 0.002) were greater in the healthy uninjured limbs compared to their VML-injured counterparts. We also found that the rehabilitation increased IGF-1 protein concentration (Main Effect; *p* = 0.018). Lastly, an analysis of MYOG revealed an interaction between treatment and rehabilitation (Interaction; *p* = 0.035).

### 2.6. Qualitative Histological Observations

**Figure 5:**
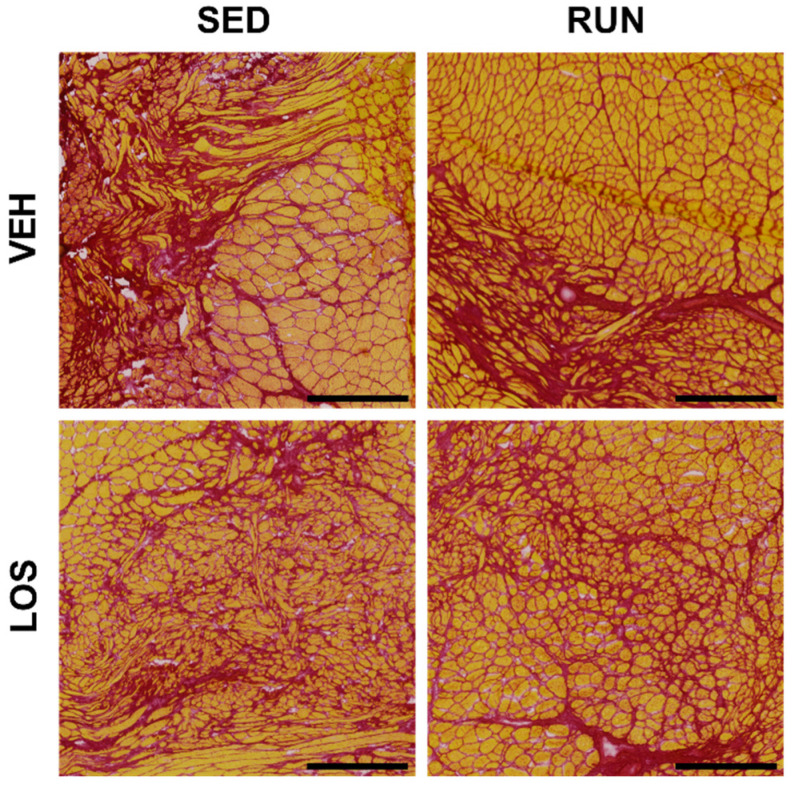
Qualitative evaluation of fibrosis via picrosirius red staining in VML-injured limbs at 56 DPI. Excessive collagen content (red stain) was observed in the defect region of the VML, indicating excessive fibrosis development in and around the site of injury for all experimental groups. Scale bars = 500 µm.

**Figure 6:**
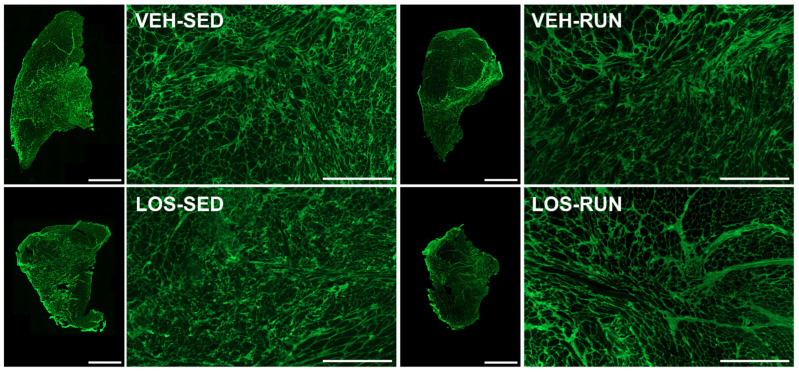
Qualitative evaluation of muscle fiber boundaries via WGA staining in VML-injured TA cross-sections at 56 DPI. All groups, regardless of antifibrotic treatment or rehabilitation, displayed similar fiber distributions with small fibers in the VML defect site and larger fibers in the uninjured regions. TA muscle cross-section scale bars = 2000 µm and VML defect site scale bars = 500 µm.

VML injuries were histologically assessed by staining muscle cross-sections for picrosirius red (PSR; Figure 5) or wheat germ agglutinin (WGA; Figure 6) at the 56 DPI endpoint. Qualitative evaluation identified excessive collagen dispersed in and around the VML defect region for all experimental groups, regardless of the losartan antifibrotic treatment. We also observed a similar distribution of small fibers intermixed with fibrous deposition within the VML region, with larger fibers in the uninjured proximal and distal regions for all groups.

### 2.7. Serum Analyte Evaluation

**Figure 7:**
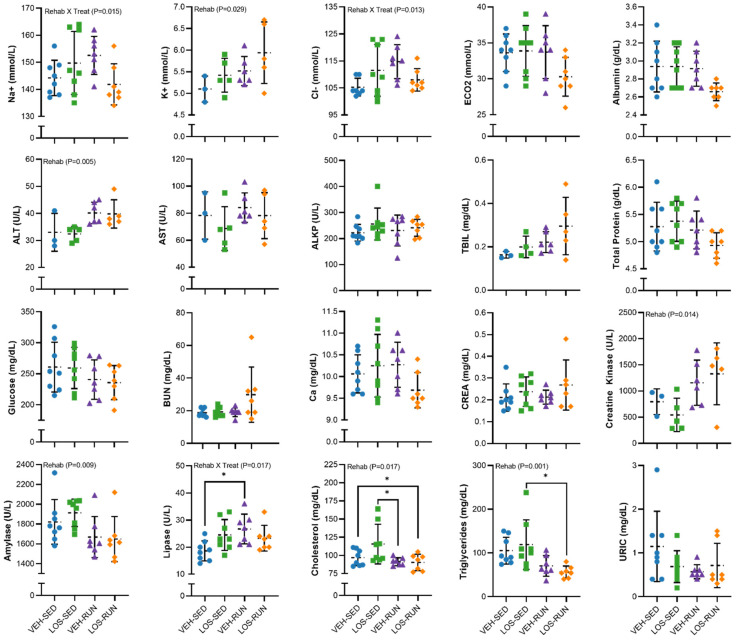
Serum metabolite evaluation at 56 days post-VML injury. Serum metabolites were analyzed for hepatic, renal, electrolyte, and additional metabolic panel markers. Data are presented as mean ± SD; n = 3–8 rats per group. Differences were considered significant when *p* < 0.05 by a two-way ANOVA followed by the Holm–Sidak post hoc test. The * indicates a significant difference of *p* < 0.05.

Serum analytes were evaluated at 56 DPI for electrolyte, hepatic, renal, and additional metabolic panel markers (Figure 7). The analysis of electrolytes revealed an effect of rehabilitation (*p* = 0.029) on serum potassium, such that the running group levels were elevated compared to sedentary groups. In addition, an interaction between the treatment and rehabilitation was observed for sodium (*p* = 0.015) and chloride (*p* = 0.013) electrolytes. The evaluation of hepatic markers found an effect of rehabilitation for alanine transaminase (ALT; *p* = 0.005); however, no effect was observed for renal analyte markers. A further analysis of pancreatic analytes identified an effect of rehabilitation for amylase (*p* = 0.009), where the running group levels were lower compared to sedentary groups. Additionally, an interaction was found between rehabilitation and antifibrotic treatment for lipase (*p* = 0.017). We also found an effect of rehabilitation on cholesterol (*p* = 0.017) and triglyceride levels (*p* = 0.001). Likewise, creatine kinase levels were increased in the running groups compared to sedentary groups (Main Effect; *p* = 0.014).

## 3. Discussion

We hypothesized that damping the fibrotic processes via losartan treatment would create a favorable microenvironment for an MMG implant to promote robust skeletal muscle regeneration and that voluntary wheel running would have a synergistic effect on the MMG-mediated wound healing response, leading to an improved muscle function output. However, our results demonstrated that the losartan treatment, which had antifibrotic properties at 28 DPI in untreated VML injuries, did not reduce fibrotic deposition when administered with MMG-treated injuries at 56 DPI, and that combining losartan treatment with voluntary wheel running actually had a detrimental effect on muscle function in VML-injured limbs.

Garg et al. (2014) demonstrated that losartan, when delivered through drinking water for 28 days post-VML injury, reduced fibrosis through the modulation of gene and protein expression [6]. In line with the previous study findings, we demonstrated a reduction in fibrosis through decreased TGFβ gene expression and decreased COL1A1 protein levels in untreated VML injuries at 28 DPI, although this effect did not persist at the more chronic timepoint of 56 days post-VML. It is well established that the defect region of untreated VML injuries will become overwhelmed by extracellular matrix (ECM) accumulation at long-term timepoints (>56 days), leading to excessive fibrotic scarring and reduced muscle function [40]. We expect that the antifibrotic effect of losartan became damped over time by the fibrotic signaling cascade, thus rendering it ineffective for long-term chronic use. This is likely due to redundant signaling mechanisms that activate the TGFβ pathway [41,42] and continue to contribute to the intrinsic fibrotic response; thus, a single receptor target may not be sufficient to maintain reduced fibrosis in a VML injury model. Minced muscle graft repair has been demonstrated to reduce ECM deposition among regenerating myofibers following VML when coupled with rehabilitation in the form of voluntary wheel running, although this effect did not extend throughout the entirety of the injury time course nor to the remaining musculature. Additionally, MMG treatment alone (no rehabilitation or antifibrotic treatment) has been demonstrated to reduce TGFβ gene expression up to 16 weeks post-VML injury compared to untreated controls [22]. Based on these prior observations, we anticipated that a long-term antifibrotic treatment with losartan would enhance the benefits of MMG repair in a VML injury by further damping the TGFβ signaling pathway. However, our findings demonstrated that despite losartan reducing fibrosis at a shorter timepoint of 28 DPI, the beneficial antifibrotic effects are diminished by 56 DPI, and the addition of voluntary wheel running did not provide any synergistic benefit, despite the presence of MMG as a pro-regenerative therapeutic.

Voluntary wheel running has been utilized in numerous studies as a form of rehabilitation for musculoskeletal injuries, including VML [22,23,43,44]. This form of exercise was implemented as the rehabilitation strategy in the present study in an attempt to facilitate improvements in muscle function and MMG-mediated tissue regeneration based on prior studies that demonstrated a reduced ECM deposition with voluntary wheel running [22]. We observed a gradual increase in running wheel activity throughout the study, accompanied by less weight gain compared to the sedentary groups, consistent with previous reports [22,23]. Animals exhibited a decrease in running wheel activity following the 28 DPI timepoint, likely attributed to analgesic effects due to the osmotic pump exchange procedure, but this activity was restored in the following weeks. Despite the increased locomotor activity, we found no beneficial improvements in muscle function compared to sedentary groups, and even less so when combined with the losartan treatment.

Serum analytes were also measured to evaluate the systemic effects of rehabilitation or losartan treatment on renal, hepatic, electrolyte, and complete metabolic function. Unsurprisingly, rehabilitation was found to increase potassium, alanine transaminase, and creatine kinase, and decrease amylase, cholesterol, and triglyceride levels. No effects on serum analytes were observed for the losartan treatment.

We anticipated that the antifibrotic treatment would improve muscle function by creating a more favorable microenvironment for muscle fibers to repair following a VML injury through reduced fibrotic scarring and ECM deposition. However, we found that losartan treatment, regardless of voluntary wheel running, resulted in lower muscle function compared to either vehicle groups. While seemingly counterintuitive, Garg et al. (2014) made a similar observation when evaluating muscle function at the 56 DPI endpoint in VML injuries treated with losartan for 28 days [6]. Interestingly, we have previously demonstrated that intentionally promoting fibrotic deposition in VML injury improves muscle function outcomes by 56 DPI [45]. This suggests that fibrosis contributes positively towards improving muscle force output, although this approach requires further investigation into the ramifications of intentionally scarring rather than regenerating the injured muscle. Despite losartan reducing the fibrosis by 28 DPI, we did not observe the same effect by day 56. This led us to posit that the early phase of ECM deposition in the defect site is potentially critical for providing a scaffold that enables cellular infiltration and bridges force transmission with the remaining musculature, and therefore, it is a crucial step in the healing process. Moreover, muscle function was measured via in vivo neuromuscular activation, rather than direct stimulation. Therefore, it is plausible that the reduced functional output could be attributed to a lack of nerve regeneration and/or innervation. Since muscle function was significantly reduced following the chronic treatment of losartan, we expect that TGFβ, in some part, plays a role in neuromuscular junction (NMJ) function and repair following VML injury [46,47]. However, further investigation is warranted to determine the relationship, if any, between TGFβ and NMJ repair in this injury model.

To further investigate the underpinnings as to why the losartan treatment reduces muscle function, we evaluated myogenic protein levels and the histomorphometry analysis of TA cross-sections. No effects of losartan were observed on myogenic protein expression, indicating that despite having an early antifibrotic effect, the treatment did not enhance MMG-mediated myogenesis in the injured muscle. However, the voluntary wheel running rehabilitation resulted in increased IGF-1 protein in the injured limbs, but not for PAX7 or MYOG, and the uninjured limbs had increased PAX7, MHC3, and IGF-1 proteins. Despite voluntary wheel running benefiting uninjured contralateral limbs, the myogenic effect did not extend to VML injuries. This is likely due to the excessive accumulation of fibrosis in the defect region, regardless of the presence of an MMG therapeutic, leading to the disruption of the existing ECM structure and a reduction in the regenerative potential of the remaining musculature [40]. The small sample size for histological evaluation (n = 3) is a limitation of the present study, requiring further experiments to increase animal numbers for more robust quantitative analyses. With this in mind, the preliminary evaluation of muscle fiber histomorphometry was performed to assess morphologic differences in the injured TA muscles (Supplemental Figure S1). The analysis of the fiber distribution by a minimum Feret diameter identified a rightward shift in the median value for both LOS-SED and VEH-RUN groups, indicating the presence of larger diameter fibers. This preliminary finding would suggest that treatment with losartan, without voluntary wheel running, leads to a higher frequency of larger diameter fibers, although no evidence of an enhanced MMG-mediated myogenesis was observed. Interestingly, the losartan without voluntary wheel running group (LOS-SED) displayed a similar distribution of fibers to that of the vehicle with voluntary wheel running group (VEH-RUN) but exhibited a decreased maximal TA torque when evaluating muscle function. Despite these preliminary findings, further samples must be analyzed for conclusive evidence.

While these data demonstrate that long-term losartan treatment and voluntary wheel running elicits negative outcomes for muscle function in VML, much is still yet to be explored. For example, the effects of losartan and voluntary wheel running as dual adjunct interventions to MMG treatment for VML injury were evaluated chronically to the 56 DPI timepoint. However, analysis at earlier time points, such as 28 DPI, would help in determining the mechanisms and timing of how losartan affects muscle regeneration and fibrosis throughout the healing process. Likewise, later time points would also be of benefit in evaluating whether losartan enables a delayed muscle regeneration response past 56 DPI. The timing and duration of antifibrotic administration are critical components in determining the treatment strategy. In a prior study, Garg et al. (2014) investigated the optimal treatment duration for losartan and found that administration for 28 DPI sufficiently reduced fibrosis, but negatively impacted muscle function [6]. Antifibrotic treatment with losartan began at 3 DPI and continued until the study endpoint of 56 days, in combination with MMG as a primary therapeutic. However, as our results indicate, the treatment with losartan for the full study duration negatively impacted muscle function outcomes, despite the presence of an MMG or rehabilitation in the form of voluntary wheel running.

In the present study, the most salient findings were that (1) long-term systemic antifibrotic treatment, in the absence of enhanced myogenesis, is detrimental to muscle function outcomes and (2) more targeted/local antifibrotic therapies may be needed to successfully damp the excessive ECM accumulation without negative impacts on the overall contractile output. Rehabilitation has the potential to be a useful tool to enhance myogenesis in VML injuries, but careful consideration must be made when determining the timing of the implementation when used in conjunction with an antifibrotic treatment. Future work will require optimizing the timing and duration of the antifibrotic administration for VML injury, as the fibrotic response is a key component of the pathophysiology. Additionally, while voluntary wheel running has been demonstrated to be beneficial for recovery following the VML injury of the TA muscle [22,23], other studies have found negative impacts when combining running with a regenerative therapeutic [43], suggesting that other forms of rehabilitation may need to be considered, including electrical stimulation and range of motion exercises [48]. Likewise, to antifibrotic treatment strategies, the timing and intensity of rehabilitation interventions are also critical factors for optimizing functional muscle recovery.

The present study investigated the interactions of an antifibrotic treatment with a running-based rehabilitation strategy on the efficacy of an MMG treatment to the VML injury. Herein, we demonstrated that chronic losartan administration, regardless of the presence of a rehabilitative intervention, decreases muscle function outcomes by 56 DPI, while no evidence of an enhanced myogenic protein expression was found for either the losartan-treated or voluntary wheel running VML injured groups. Taken together, our findings demonstrate that the use of losartan as an antifibrotic therapy in combination with an MMG treatment, regardless of the presence of concurrent running-based rehabilitation, has a negative effect on long-term muscle function and fails to enhance MMG-mediated myogenesis following VML injury.

## 4. Materials and Methods

### 4.1. Losartan Delivery Evaluation

Adult male Lewis rats (361 ± 19.8 g; Charles River Laboratories; Wilmington, MA, USA) were subjected to a well-established VML injury in the TA muscle [49]. Three days later, an osmotic pump filled with either 10 mg/kg/day losartan potassium (LOS; n = 5) or vehicle (VEH; n = 5) was implanted subcutaneously. Animals were sacrificed 28 days post-injury to determine whether subcutaneous administration of losartan potassium reduces fibrotic markers in VML injured limbs. TA muscles were harvested and subsequently crushed into powder for either pro-fibrotic gene expression (Section 4.8) or collagen protein analysis (Section 4.7).

### 4.2. Experimental Design

Adult male Lewis rats (343 ± 25.1 g; Charles River Laboratories; Wilmington, MA, USA) were subjected to a VML injury in the TA muscle and all animals were acutely treated with MMG as a primary (pro-regenerative) intervention. Animals were then randomly assigned to one of four treatment conditions: (1) vehicle treatment without rehabilitation (VEH-SED; n = 8), (2) losartan treatment without rehabilitation (LOS-SED; n = 8), (3) vehicle treatment with rehabilitation (VEH-RUN; n = 8), or (4) losartan treatment with rehabilitation (i.e., sedentary) (LOS-RUN; n = 8). The in vivo neuromuscular functional capacity was assessed in the injured and contralateral uninjured limbs at the study endpoint of 56 days post-VML injury. Animals were subsequently euthanized with a lethal dose of Euthasol (Virbac AH Inc.; Westlake, TX, USA) delivered via intracardiac injection. The total number of animals in each group was randomly divided for either molecular or histological analyses following euthanasia. TA and EDL muscles from injured and uninjured hindlimbs were harvested and weighed for gross anatomy measurements. Thereafter, the TA muscles were either flash frozen for histology or crushed into powder for gene and protein analysis. All animals were exposed to a 12-h light/dark cycle and had ad libitum access to food and water. All protocols and animal care guidelines were approved by the Institutional Animal Care and Use Committee at the Uniformed Services University of the Health Sciences (Protocol# SUR-19-995; USUHS; Bethesda, MD, USA). All experiments were conducted in compliance with the Animal Welfare Act, the Implementing Animal Welfare Regulations, and in accordance with the principles of the Guide for the Care and Use of Laboratory Animals.

### 4.3. VML Injury Model

The procedure for creating a unilateral defect was performed based on a well-characterized small animal model of VML in the TA muscle [49]. Prior to surgery, all animals received buprenorphine SR (1.2 mg/kg body weight) by subcutaneous injection for perioperative pain relief. Using an aseptic technique, a lateral incision was created in the TA muscle of the left hindlimb with the animal under anesthesia (1–3% isoflurane). The skin and underlying fascia layers were retracted from the muscle and a metal plate was inserted between the TA and EDL muscles. A full thickness VML defect was created by pressing a 6 mm biopsy punch into the belly of the TA muscle through to the underlying metal plate. The muscle defects were weighed (Table 1) and subsequently minced using sterile surgical scissors to create the MMG for implantation. The autologous MMGs were then placed inside the newly created VML defect of the TA muscle. Wounds were closed by suturing individual layers (i.e., fascia followed by skin) and a topical antibiotic was applied to the closure. Following surgery, all animals were monitored daily for 3 days to assess complications with wound closure, dehydration, and pain management. Due to complications following surgery, two animals died post-operatively, resulting in two fewer animals for the running rehabilitation groups (VEH-RUN, n = 1; LOS-RUN, n = 1).

### 4.4. Antifibrotic Adjunct Treatment

Losartan potassium powder (Selleck Chemicals; Houston, TX, USA) was dissolved in a 1:1 sterile solution of dimethyl sulfoxide (DMSO) and normal saline (0.9% sodium chloride), then loaded into osmotic pumps (2ML4; Alzet; Cupertino, CA, USA) for implantation. Three days following the VML injury, animals were again anesthetized and prepped for surgery, as previously described. Using a sterile aseptic technique, an incision was made in the dorsal region and a subcutaneous pocket was made by blunt dissection. A single osmotic pump loaded with either losartan potassium (10 mg/kg/day) or vehicle (1:1 solution of DMSO and normal saline) was implanted subcutaneously, then the incision was closed with skin staples (ShopMedVet; Mettawa, IL, USA). All animals were monitored daily for 3 days to assess complications with wound closure, dehydration, and pain management following surgery. Osmotic pumps were explanted after 28 days and new pumps were implanted following the same surgical and post-monitoring procedures.

### 4.5. Running Wheel Implementation

Animals in the rehabilitation groups (VEH-RUN and LOS-RUN) were housed with unrestricted access to a running wheel (ENV-042; Med Associates Inc.; Fairfax, VT, USA) and were acclimated 7 days prior to VML injury. The running wheel access was restricted for 7 days post-VML injury followed by unrestricted access until the 56-day study endpoint. Total running distances were digitally recorded using Med PC-V software (Med Associates Inc.; Fairfax, VT, USA), and the average daily running distance per animal was calculated weekly after each data collection.

### 4.6. In Vivo Neuromuscular Strength Assessments

The TA muscle isometric torque was measured in vivo in anesthetized rats (isoflurane; 1.5–2.0%) using a dual-mode muscle lever system (Model#305C-LR; Aurora Scientific Inc.; Aurora, ON, Canada) at 56 DPI. Transcutaneous needle electrodes were inserted on either side of the common peroneal nerve. The optimal stimulation voltage was determined for each animal with a series of twitch and tetanic contractions (150 Hz, 0.1 ms pulse width, 400 ms train). Then, a skin incision was made at the antero-lateral aspect of the ankle, and the synergist muscles were severed to capture isolated in vivo torque measurements [50]. The TA muscle isometric tetanic torque was measured (10–200 Hz) with the ankle at a right angle. The procedure was repeated on the contralateral uninjured limb, and the TA muscle isometric tetanic torque was measured only at 150 Hz (i.e., tetanic isometric torque). After all measurements were collected, the TA and EDL muscles were collected from each hindlimb.

### 4.7. Protein Analysis

Snap frozen whole TA muscles were crushed into powder using a mortar and pestle. Crushed muscles were then used for protein analysis at a sample size of n = 4 per group for the 56-day post-injury time point and n = 5 per group for the 28-day time point. Powdered muscle samples were suspended in T-PER™ (ThermoFisher; Waltham, MA, USA) lysis buffer and Halt™ Protease Inhibitor Cocktail 100× (Thermo Fisher Scientific Inc.; Waltham, MA, USA) at a concentration of 100 mg/mL. The suspended samples were homogenized and centrifuged at 10,000× *g* for 5 min at 4 °C. The supernatant was collected, and total protein concentration was quantified using a Pierce™ BCA Protein Assay Kit (Thermo Fisher Scientific Inc.; Waltham, MA, USA) according to the manufacturer’s instructions. Proteins were measured in muscle lysate using ELISA kits (MyBiosource Inc; San Diego, CA, USA). Proteins associated with fibrogenic processes included collagen type I alpha I (COL1a1), collagen 3 alpha I (COL3A1), and transforming growth factor beta (TGFβ). Proteins associated with myogenic processes included myogenin (MYOG), paired box 7 (PAX7), insulin-like growth factor (IGF-1), and myosin heavy chain 3 (MHC3). Protein lysates were further diluted in the T-PER™ lysis buffer as needed and added to antibody-coated plates along with standard protein concentrations. The plates were incubated and washed according to specific manufacturer instructions. A change in color appeared in the plate, and the optical density was measured at specific wavelengths using an Infinite M200 Pro spectrophotometer (Tecan; Männedorf, Switzerland). Fibrotic and myogenic protein concentrations were calculated by interpolation from the generated standard curve and normalized to the total protein concentration quantified from the BCA assay.

### 4.8. Gene Expression Analysis

RNA was isolated from the snap frozen whole TA muscles (n = 5 samples per group) collected at the 28-day time point. TA muscles were crushed using a mortar and pestle and RNA was extracted with Trizol Reagent (Invitrogen; Carlsbad, CA, USA). The RNA yield was quantified using a NanoDrop spectrometer (Nanodrop Technologies LLC; Wilmington, DE, USA) and the 260/280 optical density (OD) ratios were evaluated. The RNA (1000 ng) samples underwent a genomic DNA (gDNA) contamination removal step followed by a reverse transcription into complementary (cDNA) with the iScript gDNA Clear Synthesis Kit (Bio-Rad; Hercules, CA, USA) using an iCycler thermal cycler (Bio-Rad; Hercules, CA, USA). The primers used in this study are listed in Supplemental Table S1. Aliquots of cDNA (7.5 ng/20 µL reaction) were amplified in duplicate with 450 nM forward/reverse primers and iTaq™ Universal SYBR^®^ Green Supermix (Bio-Rad; Hercules, CA, USA) using a QuantStudio Flex 7 real-time polymerase chain reaction (PCR) system (Applied Biosystems; Foster City, CA, USA). Gene expression was analyzed by the 2(−ΔΔCT) method, where the ΔCT value was determined by normalizing data to the 18S ribosomal RNA housekeeping gene. The expression of the target genes was then normalized relative to the uninjured vehicle control limbs.

### 4.9. Tissue Processing and Staining

Whole TA muscles (n = 3–4 samples per group) collected during the tissue harvest were embedded in an optimal cutting temperature (OCT) compound (Sakura Finetek USA, Inc.; Torrance, CA, USA) and flash frozen using liquid nitrogen-chilled isopentane. Frozen muscle samples were stored at −80 °C until ready for processing. Tissues were then cryo-sectioned at −20 °C to a thickness of 7 µm and placed onto slides for histological staining. For sections stained with Picrosirius Red (Abcam; Cambridge, United Kingdom), tissues were fixed in 10% formalin (Sigma-Aldrich; St. Louis, MO, USA) and stained using the suggested protocol, then sealed with Surgipath Micromounting Medium (Leica; Wetzlar, Germany). For sections stained with Wheat Germ Agglutinin-AF488 (WGA;Thermo Fisher Scientific Inc.; Waltham, MA, USA), tissues were fixed in 4% paraformaldehyde and stained for WGA (1:200) and DAPI (1:2000), then sealed with a 1:1 solution of glycerol and phosphate-buffered saline. Tissues were imaged at 20× magnification for PSR and 10× magnification for WGA with an Axio Scan Z1 (Zeiss; Oberkochen, Germany) slide scanning microscope and acquisition parameters were standardized for all samples.

### 4.10. Serum Analyte Analysis

Serum samples from each animal (n = 3–8 per group) were isolated by centrifugation of whole blood samples acquired at the 56 DPI study endpoint. Samples were aliquoted and stored at −80 °C until ready for analysis. Analytes were detected in serum samples using the Vitros MicroSlide Assay panel (Ortho Clinical Diagnostics; Victoria, Australia) and measured by colorimetric methods using a Vitros 350 Chemistry System analyzer (Ortho Clinical Diagnostics; Victoria, Australia) according to the manufacturer’s instructions. Analyte measurements were averaged per group for the statistical analysis.

### 4.11. Statistical Analysis

Data were analyzed using the GraphPad Prism software version 9.3.1 (GraphPad; San Diego, CA, USA) and evaluated for normal distributions and equal variance prior to analysis. Dependent variables passed assumptions for the two-way and three-way analysis of variance (ANOVA). Mixed-effect models were used if any values were missing from the dataset. In the event of a significant interaction or Main Effect of treatment or rehabilitation, a Holm–Sidak’s multiple comparison test was performed. Statistical significance was achieved at an alpha of 0.05.

## Figures and Tables

**Table 1:** Gross Anatomy & Muscle Weights.

		Experimental Groups															
		VEH-SED				LOS-SED				VEH-RUN				LOS-RUN			
		Mean	±	SD	Size	Mean	±	SD	Size	Mean	±	SD	Size	Mean	±	SD	Size
Defect Weight (mg)	ns	88.6	±	7.19	N = 8	89.3	±	13.4	N = 8	89.7	±	10.6	N = 7	92.9	±	9.39	N = 7
Endpoint Body Weight (g)	†,¥	444	±	29.41	N = 8	410	±	28.8	N = 8	387	±	23.7	N = 7	366	±	15.9	N = 7
Weight Gain (g)	†,¥	93.4	±	12.8	N = 8	61.6	±	14.9	N = 8	53.3	±	25.9	N = 7	28.6	±	18.8	N = 7
Injured Limb TA Weight (mg/g BW)	ns	1.48	±	0.22	N = 8	1.18	±	0.19	N = 8	1.39	±	0.36	N = 7	1.31	±	0.09	N = 7
Uninjured Limb TA Weight (mg/g BW)	¥	1.84	±	0.09	N = 8	1.81	±	0.09	N = 8	2.01	±	0.09	N = 7	1.94	±	0.03	N = 7
Injured Limb EDL Weight (mg/g BW)	ns	0.45	±	0.05	N = 8	0.41	±	0.15	N = 8	0.39	±	0.11	N = 7	0.37	±	0.09	N = 7
Uninjured Limb EDL Weight (mg/g BW)	¥	0.42	±	0.04	N = 8	0.41	±	0.03	N = 8	0.47	±	0.04	N = 7	0.44	±	0.02	N = 7

† Indicates significant main effect as a function of Treatment by two-way ANOVA *(p* < 0.05). ¥ Indicates significant main effect as a function of Rehabilitation by two-way ANOVA (*p* < 0.05). BW = Body Weight, TA = Tibialis Anterior, EDL = Extensor Digitorum Longus, SD = Standard Deviation.

## Data Availability

The datasets used and/or analyzed during the current study are available from the corresponding authors upon a reasonable request.

## Supplementary Materials

The following supporting information can be downloaded at: https://www.mdpi.com/article/10.3390/ijms24043564/s1.

## References

[1] Grogan B.F., Hsu J.R., Consortium S.T.R. (2011). Volumetric Muscle Loss. J. Am. Acad. Orthop. Surg..

[2] Kulwatno J., Goldman S.M., Dearth C.L. (2022). Volumetric Muscle Loss: A Bibliometric Analysis of a Decade of Progress. Tissue Eng. Part B Rev..

[3] Aguilar C.A., Greising S.M., Watts A., Goldman S.M., Peragallo C., Zook C., Larouche J., Corona B.T. (2018). Multiscale Analysis of a Regenerative Therapy for Treatment of Volumetric Muscle Loss Injury. Cell Death Discov..

[4] Garg K., Ward C.L., Hurtgen B.J., Wilken J.M., Stinner D.J., Wenke J.C., Owens J.G., Corona B.T. (2015). Volumetric Muscle Loss: Persistent Functional Deficits beyond Frank Loss of Tissue. J. Orthop. Res..

[5] Corona B.T., Rivera J.C., Dalske K.A., Wenke J.C., Greising S.M. (2019). Pharmacological Mitigation of Fibrosis in a Porcine Model of Volumetric Muscle Loss Injury. Tissue Eng. Part A.

[6] Garg K., Corona B.T., Walters T.J. (2014). Losartan Administration Reduces Fibrosis but Hinders Functional Recovery after Volumetric Muscle Loss Injury. J. Appl. Physiol..

[7] Helal M.A.M., Shaheen N.E.M., Abu Zahra F.A. (2016). Immunomodulatory Capacity of the Local Mesenchymal Stem Cells Transplantation after Severe Skeletal Muscle Injury in Female Rats. Immunopharmacol. Immunotoxicol..

[8] Burks T.N., Cohn R.D. (2011). Role of TGF-β Signaling in Inherited and Acquired Myopathies. Skelet. Muscle.

[9] Miguel-Carrasco J.L., Beaumont J., San José G., Moreno M.U., López B., González A., Zalba G., Díez J., Fortuño A., Ravassa S. (2017). Mechanisms Underlying the Cardiac Antifibrotic Effects of Losartan Metabolites. Sci. Rep..

[10] Kovács M.G., Kovács Z.Z.A., Varga Z., Szűcs G., Freiwan M., Farkas K., Kővári B., Cserni G., Kriston A., Kovács F. (2021). Investigation of the Antihypertrophic and Antifibrotic Effects of Losartan in a Rat Model of Radiation-Induced Heart Disease. Int. J. Mol. Sci..

[11] Kim H., Baek C.H., Lee R.B., Chang J.W., Yang W.S., Lee S.K. (2017). Anti-Fibrotic Effect of Losartan, an Angiotensin II Receptor Blocker, Is Mediated through Inhibition of ER Stress via Up-Regulation of SIRT1, Followed by Induction of HO-1 and Thioredoxin. Int. J. Mol. Sci..

[12] Sookoian S., Fernández M.A., Castaño G. (2005). Effects of Six Months Losartan Administration on Liver Fibrosis in Chronic Hepatitis C Patients: A Pilot Study. World J. Gastroenterol..

[13] Salama Z.A., Sadek A., Abdelhady A.M., Darweesh S.K., Morsy S.A., Esmat G. (2016). Losartan May Inhibit the Progression of Liver Fibrosis in Chronic HCV Patients. Hepatobiliary Surg. Nutr..

[14] Lee E.-M., Kim D.-Y., Kim A.-Y., Lee E.-J., Kim S.-H., Lee M.-M., Sung S.-E., Park J.-K., Jeong K.-S. (2015). Chronic Effects of Losartan on the Muscles and the Serologic Profiles of Mdx Mice. Life Sci..

[15] Bedair H.S., Karthikeyan T., Quintero A., Li Y., Huard J. (2008). Angiotensin II Receptor Blockade Administered after Injury Improves Muscle Regeneration and Decreases Fibrosis in Normal Skeletal Muscle. Am. J. Sport. Med..

[16] Corona B.T., Wenke J.C., Ward C.L. (2016). Pathophysiology of Volumetric Muscle Loss Injury. Cells Tissues Organs.

[17] Krupicka J., Ceypová K., Kristenová P., Hauser T. (2008). The safety of long-term administration of losartan in current clinical practice: A non-intervention NCT-CZ 14/04/LOZ study. Vnitr. Lek..

[18] Habashi J.P., Judge D.P., Holm T.M., Cohn R.D., Cooper T.K., Myers L., Klein E.C., Liu G., Podowski M., Neptune E.R. (2006). Losartan, an AT1 Antagonist, Prevents Aortic Aneurysm in a Mouse Model of Marfan Syndrome. Science.

[19] Campistol J.M., Iñigo P., Jimenez W., Lario S., Clesca P.H., Oppenheimer F., Rivera F. (1999). Losartan Decreases Plasma Levels of TGF-Beta1 in Transplant Patients with Chronic Allograft Nephropathy. Kidney Int..

[20] Saunders D., Rose L. (2021). Regenerative Rehabilitation of Catastrophic Extremity Injury in Military Conflicts and a Review of Recent Developmental Efforts. Connect. Tissue Res..

[21] Greising S.M., Warren G.L., Southern W.M., Nichenko A.S., Qualls A.E., Corona B.T., Call J.A. (2018). Early Rehabilitation for Volumetric Muscle Loss Injury Augments Endogenous Regenerative Aspects of Muscle Strength and Oxidative Capacity. BMC Musculoskelet. Disord..

[22] Corona B.T., Garg K., Ward C.L., McDaniel J.S., Walters T.J., Rathbone C.R. (2013). Autologous Minced Muscle Grafts: A Tissue Engineering Therapy for the Volumetric Loss of Skeletal Muscle. Am. J. Physiol.-Cell Physiol..

[23] Aurora A., Garg K., Corona B.T., Walters T.J. (2014). Physical Rehabilitation Improves Muscle Function Following Volumetric Muscle Loss Injury. BMC Sport. Sci. Med. Rehabil..

[24] Quarta M., Cromie M., Chacon R., Blonigan J., Garcia V., Akimenko I., Hamer M., Paine P., Stok M., Shrager J.B. (2017). Bioengineered Constructs Combined with Exercise Enhance Stem Cell-Mediated Treatment of Volumetric Muscle Loss. Nat. Commun..

[25] Kurosaka M., Naito H., Ogura Y., Kojima A., Goto K., Katamoto S. (2009). Effects of Voluntary Wheel Running on Satellite Cells in the Rat Plantaris Muscle. J. Sport. Sci. Med..

[26] Rose L.F., Wolf E.J., Brindle T., Cernich A., Dean W.K., Dearth C.L., Grimm M., Kusiak A., Nitkin R., Potter K. (2018). The Convergence of Regenerative Medicine and Rehabilitation: Federal Perspectives. Npj Regen. Med..

[27] Greising S.M., Dearth C.L., Corona B.T. (2016). Regenerative and Rehabilitative Medicine: A Necessary Synergy for Functional Recovery from Volumetric Muscle Loss Injury. Cells Tissues Organs.

[28] Willett N.J., Boninger M.L., Miller L.J., Alvarez L., Aoyama T., Bedoni M., Brix K.A., Chisari C., Christ G., Dearth C.L. (2020). Taking the Next Steps in Regenerative Rehabilitation: Establishment of a New Interdisciplinary Field. Arch. Phys. Med. Rehabil..

[29] Corona B.T., Machingal M.A., Criswell T., Vadhavkar M., Dannahower A.C., Bergman C., Zhao W., Christ G.J. (2012). Further Development of a Tissue Engineered Muscle Repair Construct In Vitro for Enhanced Functional Recovery Following Implantation In Vivo in a Murine Model of Volumetric Muscle Loss Injury. Tissue Eng. Part A.

[30] Dziki J.L., Giglio R.M., Sicari B.M., Wang D.S., Gandhi R.M., Londono R., Dearth C.L., Badylak S.F. (2018). The Effect of Mechanical Loading Upon Extracellular Matrix Bioscaffold-Mediated Skeletal Muscle Remodeling. Tissue Eng. Part A.

[31] Grasman J.M., Do D.M., Page R.L., Pins G.D. (2015). Rapid Release of Growth Factors Regenerates Force Output in Volumetric Muscle Loss Injuries. Biomaterials.

[32] Gilbert-Honick J., Ginn B., Zhang Y., Salehi S., Wagner K.R., Mao H.-Q., Grayson W.L. (2018). Adipose-Derived Stem/Stromal Cells on Electrospun Fibrin Microfiber Bundles Enable Moderate Muscle Reconstruction in a Volumetric Muscle Loss Model. Cell Transplant..

[33] Baker H.B., Passipieri J.A., Siriwardane M., Ellenburg M.D., Vadhavkar M., Bergman C.R., Saul J.M., Tomblyn S., Burnett L., Christ G.J. (2017). Cell and Growth Factor-Loaded Keratin Hydrogels for Treatment of Volumetric Muscle Loss in a Mouse Model. Tissue Eng. Part A.

[34] Aurora A., Wrice N., Walters T.J., Christy R.J., Natesan S. (2018). A PEGylated Platelet Free Plasma Hydrogel Based Composite Scaffold Enables Stable Vascularization and Targeted Cell Delivery for Volumetric Muscle Loss. Acta Biomater..

[35] Grasman J.M., Zayas M.J., Page R.L., Pins G.D. (2015). Biomimetic Scaffolds for Regeneration of Volumetric Muscle Loss in Skeletal Muscle Injuries. Acta Biomater..

[36] Corona B.T., Henderson B.E.P., Ward C.L., Greising S.M. (2017). Contribution of Minced Muscle Graft Progenitor Cells to Muscle Fiber Formation after Volumetric Muscle Loss Injury in Wild-Type and Immune Deficient Mice. Physiol. Rep..

[37] Goldman S.M., Henderson B.E.P., Walters T.J., Corona B.T. (2018). Co-Delivery of a Laminin-111 Supplemented Hyaluronic Acid Based Hydrogel with Minced Muscle Graft in the Treatment of Volumetric Muscle Loss Injury. PLoS ONE.

[38] Greising S.M., Corona B.T., McGann C., Frankum J.K., Warren G.L. (2019). Therapeutic Approaches for Volumetric Muscle Loss Injury: A Systematic Review and Meta-Analysis. Tissue Eng. Part B Rev..

[39] Langridge B., Griffin M., Butler P.E. (2021). Regenerative Medicine for Skeletal Muscle Loss: A Review of Current Tissue Engineering Approaches. J. Mater. Sci. Mater. Med..

[40] Lieber R.L., Ward S.R. (2013). Cellular Mechanisms of Tissue Fibrosis. 4. Structural and Functional Consequences of Skeletal Muscle Fibrosis. Am. J. Physiol.-Cell Physiol..

[41] Wynn T. (2008). Cellular and Molecular Mechanisms of Fibrosis. J. Pathol..

[42] Huang F., Chen Y.-G. (2012). Regulation of TGF-β Receptor Activity. Cell Biosci..

[43] Hu C., Ayan B., Chiang G., Chan A.H.P., Rando T.A., Huang N.F. (2022). Comparative Effects of Basic Fibroblast Growth Factor Delivery or Voluntary Exercise on Muscle Regeneration after Volumetric Muscle Loss. Bioengineering.

[44] Tsai L.-C., Cooper E.S., Hetzendorfer K.M., Warren G.L., Chang Y.-H., Willett N.J. (2019). Effects of Treadmill Running and Limb Immobilization on Knee Cartilage Degeneration and Locomotor Joint Kinematics in Rats Following Knee Meniscal Transection. Osteoarthr. Cartil..

[45] Clements M.P., Byrne E., Camarillo Guerrero L.F., Cattin A.-L., Zakka L., Ashraf A., Burden J.J., Khadayate S., Lloyd A.C., Marguerat S. (2017). The Wound Microenvironment Reprograms Schwann Cells to Invasive Mesenchymal-like Cells to Drive Peripheral Nerve Regeneration. Neuron.

[46] Feng Z., Ko C.-P. (2008). Schwann Cells Promote Synaptogenesis at the Neuromuscular Junction via Transforming Growth Factor-Β1. J. Neurosci..

[47] Dolan C.P., Motherwell J.M., Franco S.R., Janakiram N.B., Valerio M.S., Goldman S.M., Dearth C.L. (2022). Evaluating the Potential Use of Functional Fibrosis to Facilitate Improved Outcomes Following Volumetric Muscle Loss Injury. Acta Biomater..

[48] Basten A.M., Raymond-Pope C.J., Hoffman D.B., Call J.A., Greising S.M. (2023). Early Initiation of Electrical Stimulation Paired with Range of Motion after a Volumetric Muscle Loss Injury Does Not Benefit Muscle Function. Exp. Physiol..

[49] Dolan C.P., Dearth C.L., Corona B.T., Goldman S.M. (2022). Retrospective Characterization of a Rat Model of Volumetric Muscle Loss. BMC Musculoskelet. Disord..

[50] Wu X., Corona B.T., Chen X., Walters T.J. (2012). A Standardized Rat Model of Volumetric Muscle Loss Injury for the Development of Tissue Engineering Therapies. BioResearch Open Access.

