# Supplementary material for: Effects of Adjunct Antifibrotic Treatment within a Regenerative Rehabilitation Paradigm for Volumetric Muscle Loss
Source: Int J Mol Sci. 2023 Feb 10;24(4):3564. doi: 10.3390/ijms24043564 (PMC9964131; doi:10.3390/ijms24043564)
Supplement: Supplementary file 1 [file ijms-24-03564-s001.zip › ijms-2139903-supplementary.pdf]

International Journal of
Molecular Sciences

MDPI

Supplemental Material

Table S1. Primer sequences used for quantitative RT-PCR.

Gene	Forward Sequence	Reverse Sequence
Ctgf	5′ – AAAGACGTTTGTGCCTATTG – 3′	5′ – ACAGTCACTCAGGTTACAG – 3′
Tgfβ	5′ – GGAAATCAATGGGATCAGTC – 3′	5′ – CTGAAGCAGTAGTTGGTATC – 3′
Col3a1	5′ – CAGAACATTACATACCACTGC – 3′	5′ – CCATTCACCAGTGTGTTTAG – 3′
Col1a1	5′ – TGGATTCCAGTTCGAGTATG – 3′	5′ – AGTGATAGGTGATGTTCTGG – 3′
Acta2	5′ – AAACCACCTATAACAGCATC – 3′	5′ – AGACAGAATATTTGCGTTCTG – 3′
18S	5′ – GGAGGTAGTGACGAAAAATAACAAT– 3′	5′ – TTGCCCTCCAATGGATCCT– 3′

Connective Tissue Growth Factor (Ctgf); Transforming Growth Factor-β (Tgfβ); Collagen Type 3 Alpha 1 (Col3a1); Collagen Type 1 Alpha 1 (Col1a1); Smooth Muscle Actin Alpha 2 (Acta2); 18S ribosomal RNA (18S).

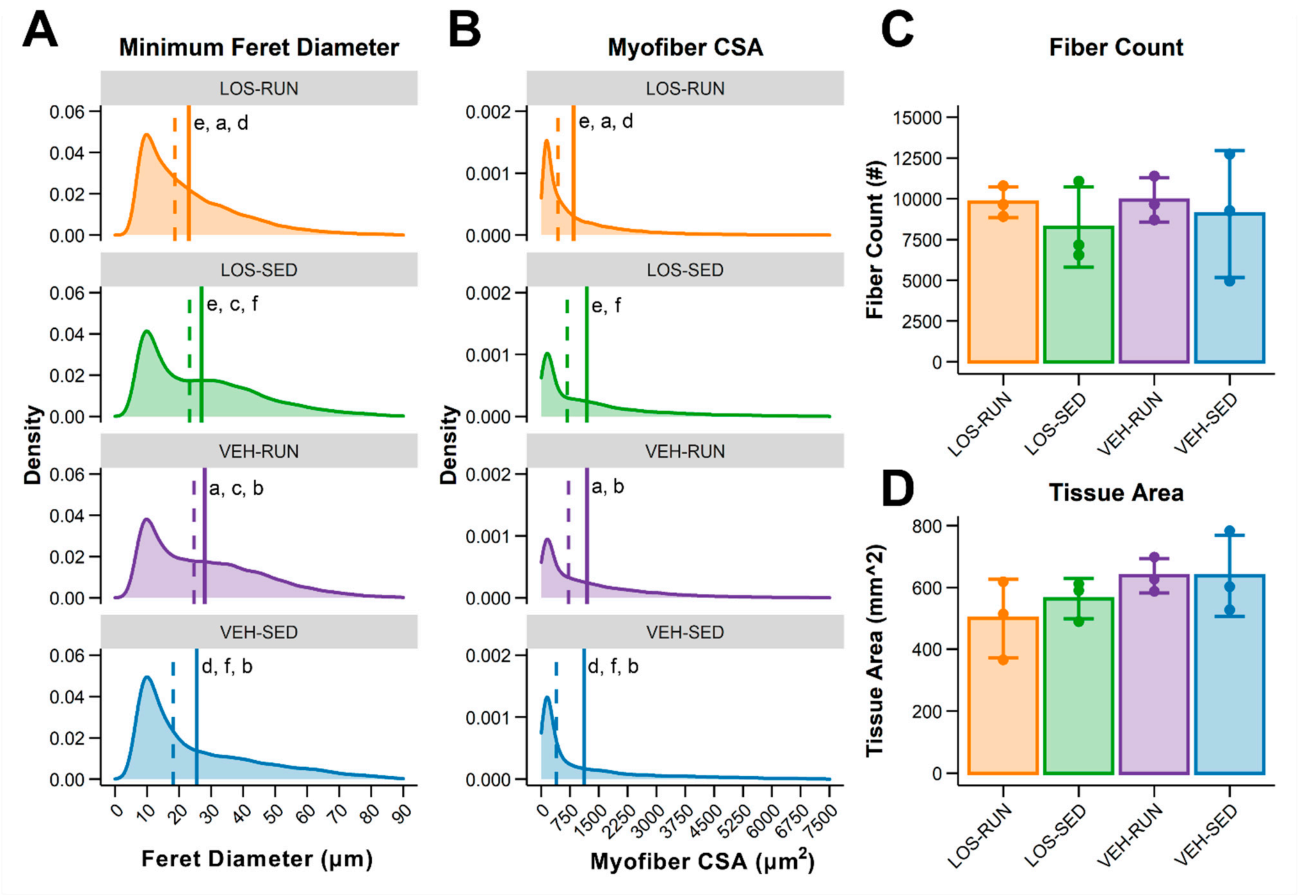

Figure S1. Muscle fiber morphology analysis. (**A**) Kernel density estimation of the minimum Feret diameter distribution for each group. The dashed line represents the median value and the solid line represents the mean value. Data are presented as the cumulative distribution from n = 3 rats per group. (**B**) Kernel density estimation of myofiber CSA distribution for each group. The dashed line represents the median value and the solid line represents the mean value. Data are presented as the cumulative distribution from n = 3 rats per group. (**C**) Evaluation of the average myofiber count per group, where the individual data points represent the myofiber count per muscle per rat. Data are presented as mean ± SD; n = 3 rats per group. (**D**) Evaluation of the average tissue cross-sectional area (CSA) per group, where the individual data points represent the tissue CSA per muscle per rat. Data are presented as mean ± SD; n = 3 rats per group. Differences were considered significant when $p < 0.05$ by a Two-Way ANOVA followed by the Holm–Sidak post hoc test. Pairwise comparisons are annotated according to the following: a = VEH-RUN to LOS-RUN; b = VEH-RUN to VEH-SED;

c = VEH-RUN to LOS-SED; d = LOS-RUN to VEH-SED; e = LOS-RUN to LOS-SED; f = VEH-SED to LOS-SED.

Disclaimer/Publisher's Note: The statements, opinions and data contained in all publications are solely those of the individual author(s) and contributor(s) and not of MDPI and/or the editor(s). MDPI and/or the editor(s) disclaim responsibility for any injury to people or property resulting from any ideas, methods, instructions or products referred to in the content.
